# Distribution and determinants of COVID-19 seroprevalence in a hard-to-access health district in Mali

**DOI:** 10.1371/journal.pgph.0004842

**Published:** 2025-07-21

**Authors:** Sekou Oumarou Thera, Mady Cissoko, Jordi Landier, Zoumana Doumbia, Amagoron Mathias Dolo, Siriman Traore, Abdoul Karim Sangare, Ibrahima Berthe, Ismaila Thera, Hadiata Berthe, Elisabeth Sogodogo, Karyn Coulibaly, Abdoulaye Guindo, Hubert Balique, Souleymane Sanogo, Charles Dara, Flore-Apolline Roy, Issaka Sagara, Bourema Kouriba, Abdoulaye A. Djimdé, Luis Sagaon-Teyssier, Laurent Vidal, Marc-Karim Bendiane, Jean Gaudart

**Affiliations:** 1 Aix Marseille Univ, IRD, INSERM, SESSTIM, ISSPAM, Marseille, France; 2 Malaria Research and Training Center (MRTC), FMOS & FAPH, USTTB, Bamako, Mali; 3 Tombouctou Regional Health Department, Tombouctou, Mali; 4 Charles Mérieux Infectiology Center, Bamako, Mali; 5 Tombouctou Hospital, Tombouctou, Mali; 6 Health and Public Hygiene Department, Bamako, Mali; 7 Aix Marseille Univ, IRD, INSERM, SESSTIM, ISSPAM, AP-HM, Hop. La Timone, BioSTIC, Marseille, France; Human Sciences Research Council, SOUTH AFRICA

## Abstract

In November 2023, three years after the onset of the pandemic, Mali officially recorded 33,160 confirmed COVID-19 cases and 743 related deaths. Tombouctou health district, following Bamako, the capital city, emerged as the second major hotspot with over 532 confirmed cases. However, these figures likely underestimate the true scale of the epidemic due to limited healthcare access and diagnostic capacity. This study aimed to describe the early trajectory of the epidemic, estimate seroprevalence, and identify factors associated with COVID-19 in the Tombouctou health district. A multi-level study was conducted in January 2021. First, the epidemic dynamics in Tombouctou were analyzed, estimating the basic reproduction number (R0) using daily case time series. Additionally, a cross-sectional survey was conducted, involving 419 households and 1102 participants, allowing for seroprevalence estimation by age and gender. A face-to-face questionnaire collected information on living conditions and knowledge, attitudes, behaviors and practices, regarding the epidemic. Factors associated with SARS-CoV-2 seropositivity were determined using generalized additive mixed models (GAMMs), adjusted to the variable under study and the level of analysis. We estimated an R0 of 2.08 [1.46-2.93]. The crude seroprevalence of SARS-CoV-2 was 33.5% (309/923, 95% CI: 30.4% to 36.6%). Living in a household where someone had been diagnosed with COVID-19 [Incidence Rate Ratio (IRR)=5.47; 95% CI (4.51 to 6.64)], traditionally wealth households [IRR = 2.02; 95% CI (1.23–3.33)], modernly wealth households [IRR = 1.33; 95% CI (1.02 – 1.72)], and older age (per year) [Adjusted Odds Ratio (AOR)=1.02 (1.00 to 1.03)] were significantly associated with seropositivity. Our analysis highlighted the active circulation of SARS-CoV-2 in Tombouctou, with higher seroprevalence observed among people from wealth households, as well as older age groups. The findings underscore the need for tailored and targeted approach focusing on specific households, demographics and settings.

## 1. Background

In November 2019, a highly contagious and infectious disease caused by the severe acute respiratory syndrome coronavirus 2 (SARS-CoV-2), named COVID-19, emerged from Wuhan, China. This outbreak quickly spread worldwide and was declared a “pandemic” by the World Health Organization (WHO) on March 11, 2020 [1]. COVID-19 is the third major coronavirus outbreak, which has proven to be the deadliest of all. By August 2023, COVID-19 had resulted in 769.77 million infections globally, with 6.95 million deaths [2].

Although the WHO African region appears being less affected since the beginning of the pandemic, the true extent of SARS-CoV-2 infection in many African countries could be underestimated [3]. This underreporting is possibly due to asymptomatic or mildly symptomatic infections, a reluctance to seek medical consultation, as well as limited access to healthcare and diagnostic capabilities [4].

Following the outbreak of the pandemic, Mali implemented a national response strategy on March 18, 2020 [5], based on the existing epidemiological information system and utilizing the District Health Information Software version 2 (DHIS2) for other epidemic diseases such as malaria, focused on district chief medical officers and regional and national hospitals, responsible for detecting (and reporting) clinical cases and establishing containment measures. In Mali, the first reported case was around March 25, 2020 [6]. This case involved a 65-year-old man arriving in Bamako from Paris, France, on March 12, 2020, who then traveled from Bamako to Kayes on March 15, 2020, where he passed away on April 5 after being hospitalized. Since then, the country experienced a rapid spread of COVID-19. From January 3, 2020, to November 22, 2023, 33,160 confirmed cases and 743 deaths have been reported in the country [7]. All health districts (HDs) have been affected, with varying levels of transmission, higher in large cities like Bamako, Kayes, and Tombouctou, where mobility is greater compared to rural areas. It can be observed that mainly cases recorded were in Greater Bamako (64.8%), and the second hotspot with the highest number of cases was the Tombouctou health district (HD), with over 532 confirmed cases (representing 0.76% of the general population) and 9 deaths.

The Tombouctou HD is one of the HDs in Mali, located in the northern region, facing political insecurity since 2012 [8]. The first case recorded in the Tombouctou region involved a MINUSMA officer, diagnosed around April 3 and hospitalized in Bamako [9]. No secondary cases were subsequently reported. The first case within the urban community of Tombouctou was reported in May: a 42-year-old jeweler, with a history of diabetes but no recent travel, first sought care for fever and symptoms of polyuria and polydipsia at a private clinic on May 2. Malaria and diabetic imbalance were initially diagnosed and treated. The patient was then transferred on May 6 to the regional hospital due to severe coughing and dyspnea, where he was managed by an infectious disease specialist with experience from the 2017 Ebola outbreak. A nasopharyngeal swab performed on May 6 returned positive for COVID-19 (result received May 8), and the patient passed away on May 8. This first reported case was likely not the index case, as four other cases were reported on May 8, marking the start of the epidemic in this northern region. Since then, Tombouctou became the second most affected region in terms of case numbers after Bamako [10]. The description of these initial cases was based on official national and regional reports as well as health facility records from Tombouctou. Apart from these partial reports, Tombouctou did not have any situational analysis report for COVID-19. In response to the epidemic surge, the strategy was revised. First, on May 30, 2020, a mobile laboratory from the Charles Merieux Infectiology Center (CMIC, Bamako) arrived in Tombouctou to expedite biological diagnostics. Subsequently, all contacts were tested, including asymptomatic individuals. All symptomatic positive patients were hospitalized; asymptomatic positive cases were monitored at home (with isolation, information provided, and distribution of masks) until they tested negative (on day 7 or day 12); negative individuals were neither monitored nor subjected to a secondary test.

Similar to seroprevalence studies in other parts of the country showing higher virus circulation than what has been reported [6,10,11], it is possible that many cases have also gone undertected in Tombouctou HD. Moreover, insecurity, the weakness of the healthcare system, and a low economic level reinforce this hypothesis. Similar situations have been documented in other countries affected by armed conflict, notably Libya, Syria, Yemen, Afghanistan, where prolonged internal strife has severely disrupted health infrastructures, hindered early case detection, and obscured the true progression of the pandemic, likely resulting in underestimating the number of infections [12–16]. A cross-sectional seroprevalence study was then conducted in Tombouctou HD to estimate the actual number of people infected with COVID-19.

Several studies have shown that the incidence and risk of COVID-19 vary in space and time and are strongly correlated with socio-economic and demographic determinants [17–19]. In Iran in 2020, Ramírez-Aldana et al. studied the spatial distribution of COVID-19 cases and identified significant spatial clusters of cases, as well as the effect of the socio-economic characteristics of Iranian provinces on the number of cases [20]. In 2021 in the United States, Islam et al. reported a positive association between COVID-19 mortality and social factors [21]. Fielding-Miller et al. (2020) found that a higher percentage of people living at or below the poverty line in non-urban areas are at increased risk of COVID-19 mortality [22]. These studies have been conducted in developed countries. Studies on the spatial distribution of COVID-19 are still limited in number and scope in developing countries, particularly in Mali.

Using data collected since the first confirmed cases of COVID-19, this study explored the spatial distribution and determinants of COVID-19 seroprevalence in Tombouctou HD. The general objective of our work was to understand the dynamics of the COVID-19 epidemic within the Tombouctou HD, and more specifically to: 1) describe the initial evolution of the epidemic using case reporting, 2) estimate the COVID-19 seroprevalence within the population, 3) identify the social, economic, and behavioral conditions associated with COVID-19 seropositivity within the population, and 4) analyze the spatial distribution of COVID-19 seroprevalence.

## 2. Materials and methods

### 2.1 Ethics statement

Authorization to conduct this study was obtained from the Ministry of Health and Social Affairs of Mali (National Coordination for COVID-19 Response) on August 28, 2020 (decision letter number 2020–001424-MSAS-SG), along with approval from the ethics committee of the Faculties of Medicine and Odonto-Stomatology and Pharmacy, University of Sciences, Technics and Technologies of Bamako (Mali), obtained on August 10, 2020, under number [2020/162/CE/FMOS/FAPH]. After explaining and discussing the study protocol, community approval was obtained from the HD chief medical officer, local religious leaders, community associations, and municipal authorities. Written consent and/or assent from the participant or their parent/guardian was also obtained before data collection. Consent forms was administered in French and local languages for better understanding. For participants or guardians who were illiterate, verbal consent was obtained in the presence of a literate, witness who documented and signed the consent process on behalf of the participant or guardian. For minor participants, assent was obtained from the minors themselves, along with written consent from their parent or legal guardian. Consent was not waived for this study, all participants or their guardians provided explicit consent prior to data collection.

### 2.2 Study design

**Fig 1:**
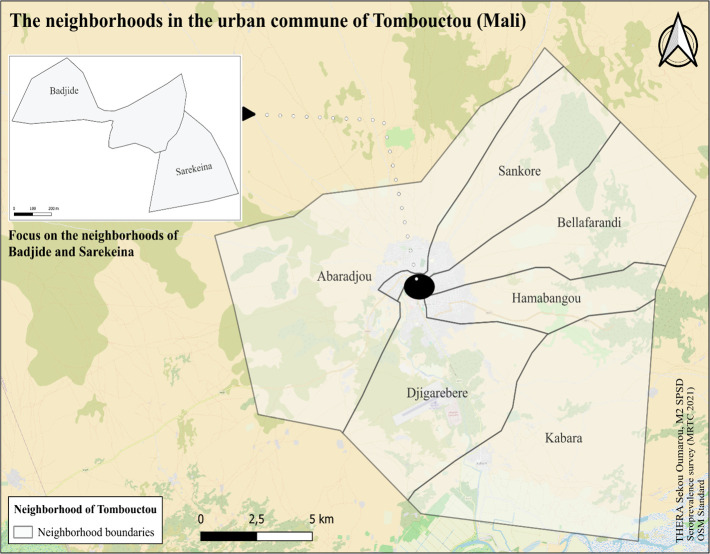
The neighborhoods in the urban commune of Tombouctou where the survey was conducted.

In accordance with the WHO guidelines protocol for age-stratified population-based seroepidemiological surveys for COVID-19 infection, a cross-sectional household survey was conducted [23] in the Tombouctou HD [Tombouctou urban commune (Fig 1)] in January 2021. At the time of the study, the number of reported cases in the Tombouctou urban commune was 532 cases across the eight neighborhoods.

Furthermore, using time series data of daily cases, we analyzed the dynamics of the epidemic in Tombouctou by estimating the basic reproduction number (R0).

At the outset of an infectious disease epidemic, an immediate objective is to determine the basic reproduction number (R0) in order to inform public health decision-makers and take the necessary measures to mitigate its spread [24]. R0 is defined as the average number of secondary cases generated by one contagious infected case [25]. The value of R0 provides information on the status of the epidemic: if R0 < 1, the epidemic will disappear, whereas if R0 > 1, the epidemic may spread within the population. Various methods have been used to estimate the R0 of the COVID-19 epidemic [26–28]. In our study, we estimated it using the time series of daily cases, according to equation 1:


$${\left[R0\right]}^{-1}=\;\int_{t=0}^{gp}\left[e\{-r\times t\}\times GI(t)d(t)\right]$$ (1)

Where t is the date of the first reported case, GI is the distribution of the generation interval, following a Gamma Γ {7; 5.2} distribution. The exponential growth period (gp) was estimated using a quasi-Poisson regression model [log (Cases)=f(time)] and the exponential growth rate (r) was extracted as a coefficient of the quasi-Poisson regression model. The time series of reported cases was carried out on a daily basis for Tombouctou (S1 Data). We considered the time series over a one-year period, from April 04, 2020 to April 04, 2021. The population was estimated on the basis of the 2020 census published by the Institut National de la Statistique (INSTAT) du Mali, using a growth rate of 2.8% [29].

### 2.3 Methods and sample size calculation

A multi-stage cluster sampling method covering all age groups one year and older in the population was conducted [30]. First, the sample size was proportional to the population size of each neighborhood. Next, each neighborhood was divided into at least four sectors of relatively equal size in terms of population distribution. The household survey thus involved each sector of the neighborhood. The first household in each sector was selected by choosing a random direction from the center, counting the houses along that street, and selecting one at random. Subsequent households were chosen by visiting the nearest house to the previous one. All household members in the age group who consented to participate were recruited.

The study was conducted among the general population aged at least one year for the seroprevalence survey and at least 12 years for the survey on knowledge, attitudes, behaviors, and practices (KABP).

The sample size was calculated assuming an observed prevalence of COVID-19 infection of 0.07% (0.07 cases/100 inhabitants) within the population of Tombouctou. Based on this assumption, a sample size of 1114 participants was estimated, with a precision of 2% and a 95% confidence interval (CI).

### 2.4 Data collection

After obtaining informed consent from the participants or their parents (or legal guardians), 2 mL of blood was drawn from all willing participants via venipuncture to conduct serological tests. Following the blood draw, a face-to-face questionnaire was administered to collect information on socio-demographic factors, including age, sex, profession, education level, and socio-economic status. The questionnaire also included items (questions) related to personal KABP concerning health associated with COVID-19.

#### 2.4.1 Laboratory methods.

The level of exposure of the population to COVID-19 was estimated through serology. Serological tests were performed on blood samples. Specific IgM and IgG antibodies against SARS-CoV-2 were measured in the serum using the VIDAS anti-SARS-CoV-2 IgM and anti-SARS-CoV-2 IgG kits (BioMerieux, Lyon, France) [31]. The VIDAS anti-SARS-CoV-2 IgM and anti-SARS-CoV-2 IgG tests aim to measure the presence of antibodies in individuals infected with SARS-CoV-2 in less than 30 minutes. Serological analyses were conducted at the Charles Mérieux Infectiology Center in Bamako, Mali.

Participants were defined as seropositive for SARS-CoV-2 if they tested positive for IgG, IgM, or both. Participants were defined as seronegative for SARS-CoV-2 if they tested negative for both IgG and IgM. Participants for whom the results could not be interpreted (uninterpretable) were excluded from the seroprevalence analysis.

#### 2.4.2 Household living conditions.

The head of the household was invited to respond to a specific questionnaire regarding the socio-cultural characteristics of the household (number of people living in the household, housing conditions, modern goods and amenities, income, religion, and the primary language spoken by members), including personal characteristics of the head of the household (education and profession). The geographic coordinates of each household participating in the study were also obtained.

By assessing the characteristics of household living conditions, three specific profiles were determined by using Hierarchical Ascendant Classification on multiple correspondence analysis (see below): **“**Traditionally Wealth Households (TWH)”, “Low-Wealth Households (LWH)”, “Modernly Wealth Households (MWH)”. Household wealth was defined as the total of goods and amenities, either material or symbolic, either marketable or not, possessed by a household and shared by household members [32]. To better reflect the diversity of local socio-economic realities, we defined household profiles based on two dimensions of wealth: traditional wealth and modern wealth. Traditional wealth was primarily based on indicators such as livestock ownership and household size, while modern wealth was assessed through the possession of modern goods and amenities (television, electricity, access to running water at home, housing size...). For each household, profiles were assigned to all members.

#### 2.4.3 Knowledge, Attitudes, Behaviors, and Practices (KABP).

To evaluate the participants’ knowledge about COVID-19, we used a scoring scale based on 13 questions (true/false/don’t know) covering prevention, treatment, symptoms, and transmission of SARS-CoV-2. We also assessed the participants’ opinions on the disease using 4 bipolar Likert questions (ranging from strongly agree to strongly disagree) focusing on the origin of the infection: a divine punishment, witchcraft, a “White people’s” disease, and a means for the rich to make money.

Changes in participants’ behaviors since the onset of the epidemic were measured using 6 bipolar Likert questions (ranging from systematically/often to never) related to: handwashing, visiting sacred areas, visiting friends and family, touching others, and sneezing into one’s elbow. Risky practices were assessed with 4 bipolar Likert questions (ranging from systematically/often to never) concerning behaviors in the past seven days: wearing a mask outside the home, spending more than 2 hours in a closed space with others, visiting crowded places during the day (bus stations, markets, etc.) and at night (bars, nightclubs, restaurants).

By assessing the characteristics related to knowledge, attitudes, behaviors, and practices, three behavioral profiles were determined by using Hierarchical Ascendant Classification on multiple correspondence analysis (see below): “Poorly Informed and not Proactive in prevention (PIP)”, “Moderately Informed and Moderately cautious (MIM)”, “Informed and cautious but with a Negative Opinion (INO)”. These profiles grouped individuals based on their levels of KABP related to COVID-19.

### 2.5 Measures

#### 2.5.1 Outcome variable.

The outcome variable in this study differed depending on the level of analysis:

1) At the household level, the outcome was the number of seropositive cases within the household, a count type variable (positive integer).
2) At the individual level, the outcome was the SARS-CoV-2 serological status, a binary variable distinguishing participants who were seropositive from those who were seronegative.

#### 2.5.2 Independent variables.

The independent variables included in this study varied according to the level of analysis:

1) At the household level, the independent variables were:

Household living condition profiles, a categorical variable with three modalities (TWH/LWH/MWH);
Intra-household contamination: a binary variable (yes/no) indicating the presence of more than one SARS-CoV-2 seropositive case within the household.

2) At the individual level, the independent variables were:

Age: included as a continuous variable in regression models and categorized into age groups for descriptive analyses;
Sex: a binary variable (male/female);
KABP profiles: a categorical variable with three modalities (PIP/MIM/ INO).

### 2.6 Data analysis

A descriptive analysis of the data was performed using mean values, prevalence, and frequencies with 95% CI. The main statistical tests were conducted using a significance threshold of α = 5%, but with a Bonferroni correction for subgroup analyses. Proportions were compared using Fisher’s exact test. Means were compared using the Wilcoxon-Mann-Whitney test. Models outcomes were expressed as Incidence Rate Ratios (IRR) or Adjusted Odds Ratios (AOR), depending on the type of outcome variable and the level of analysis.

The seroprevalence (in %) was estimated by taking the number of SARS-CoV-2 positive individuals among all samples. We estimated the number of cases that occurred between the beginning of the epidemic and the time of the survey (January 2021) using the population of Tombouctou by sex and age categories. We approximated the number of deaths using the reported case fatality rates by age group and sex from the early days of the pandemic (February-March in China, before clinical management was optimized) [33].

Household profiles and KABP profiles were determined using a two-step descriptive approach [34]: first, a multiple correspondence analysis (MCA), followed by hierarchical Ascendant classification (HAC). This approach led to the identification of profiles differentiated by living conditions characteristics (household profile) and their KABP.

To analyze the factors associated with seropositivity to SARS-CoV-2, we used generalized additive models (GAM) [35] that varied based on the outcome variable and the level studied:

1) At the household level, we applied a quasi-Poisson regression model. The outcome variable was the number of seropositive cases in the household. We examined the effects of household profiles, as well as intra-household contamination. To account for the household structure and spatial autocorrelation, we included both an offset function based on household size and Gaussian Process (GP) based on geographical coordinates in the model. Thus, the model used was formulated as follows:

$$\begin{array}{cc} Log\;(number\;of\;positive\;cases)\; & =\;\beta 0\;+\;\beta 1(household\;profiles)\;+\;\beta 2(intra-household\;contamination)\;\\ & +\;offset\;(log(household\;size))\;+\;GP(Long,\;Lat) \end{array}$$ (2)

2) At the individual level, a generalized additive logistic mixed model (GAMM) [36] was used. The variable outcome was the SARS-CoV-2 serological status (serology negative or positive). We analyzed the effects of age, sex, and KABP profiles. To account for the household structure and spatial autocorrelation, we included both a random effect (re) for the household and Gaussian Process (GP) based on geographical coordinates in the model. Thus, the model used was formulated as follows:

Serology = β0 + β1(Ag)+ β2(Sex)+ β3(KABP profils)+ re(household)+ GP(Long, Lat) (3)

A spatial analysis was conducted to explore the geospatial distribution of COVID-19 seroprevalence in Tombouctou, as well as household and KABP profiles. The base map of Tombouctou’s neighborhoods was generated using participants’ GPS coordinates, from which an average coordinate was calculated for each neighborhood. Neighborhood boundaries were then delineated using the Voronoi polygon method based on these averaged geographic coordinates [37]. A choropleth map using natural break points (Jenks method) was created to visualize seroprevalence across different neighborhoods. The distribution of household and KABP profiles was illustrated through pie charts showing the proportion (%) of each profile within neighborhoods. Complementing these maps, we measured spatial autocorrelation of COVID-19 positivity within households. For this, we applied Martin Kulldorff’s SaTScan elliptical scan method [38]. This method allowed us to identify a high-risk cluster of COVID-19 positivity during the study period, calculating a relative risk (RR) and p-value.

All data analyses were performed using R software version 4.3.3 and SaTScan version 9.7. We used specific packages such as gtsummary, FactoMineR, lme4, gamm4. Maps were produced using QGIS software version 3.36.2.

### 2.7 Inclusivity in global research

Additional information regarding the ethical, cultural, and scientific considerations specific to inclusivity in global research is included in the supporting information (S1 Checklist).

## 3. Results

### 3.1 R0 Estimation

**Fig 2:**
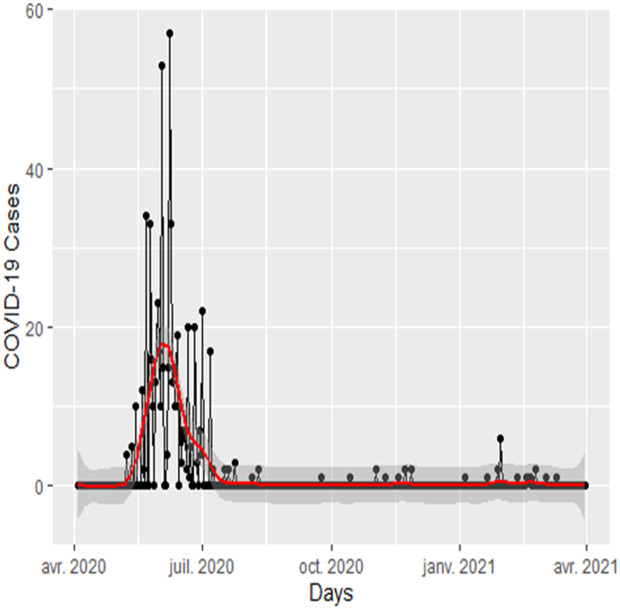
Epidemic curve of COVID-19 in Tombouctou, from the start of the epidemic (April 2020).

Analyzing data from the 598 cases during the first phase of the epidemic (Fig 2), we estimated an R0 of 2.08 [95% CI: 1.46–2.93]. This high value (R0 = 2.08 > 1) indicates the rapid spread of the infection within the Tombouctou population.

To April 2021 (one year). The black curve and points correspond to confirmed and reported cases, with or without symptoms. The red curve represents the smoothed number of cases (loess smoothing function), along with the 95% confidence interval (gray).

### 3.2 Description of the study population

**Fig 3:**
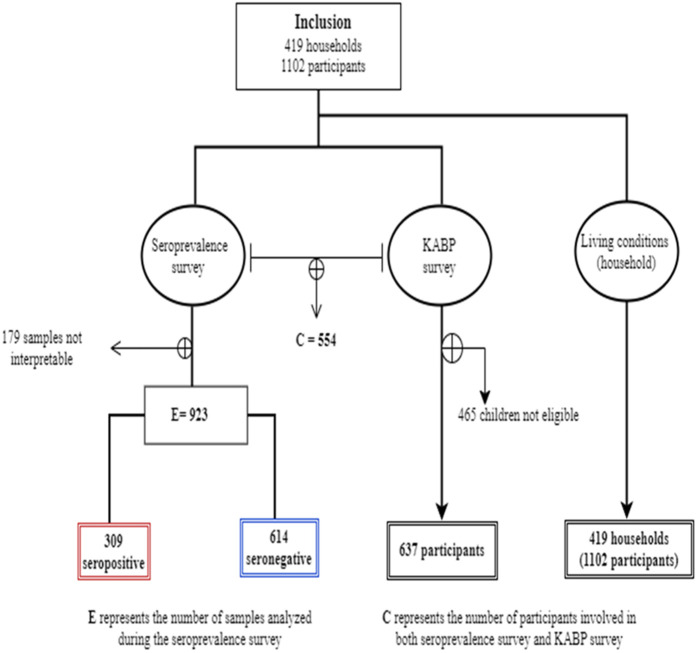
Flow chart of the study (seroprevalence survey and KABP survey).

A total of 419 households comprising 1102 individuals (S2 Data) across 8 neighborhoods were included in the study (Fig 3).

Among the 1102 participants, 923 (or 83.7%) aged at least 1 year provided a blood sample and were analyzed for COVID-19 serology (seroprevalence survey). Additionally, 637 participants (or 57.8%) aged at least 12 years responded to the questionnaire regarding personal health KABP related to COVID-19 (KABP survey). Among the 923 participants with serological results (S1 Table), 554 also have data from the KABP survey. Data on living conditions, collected from the household head, were obtained for all 419 households.

The majority of the study participants were women, accounting for 61% (673/1102). The most represented age groups were primarily young individuals: 26% were under 10 years old, 23% were aged 10–20 years, and 17% were between 20 and 30 years old (Table 1).

**Table 1:** Socio-demographic characteristics of study participants (N = 1102).

Variables	n	% (95% CI)
**Site (Neighborhood)**		
Abaradjou	267	**24 (22-27)**
Badjide	62	6 (4-7)
Bellafarandi	135	12 (10-14)
Djigarebere	112	10 (8-12)
Hamabangou	222	**20 (18-23)**
Kabara	132	12 (10-14)
Sankore	125	11 (9-13)
Sarekeina	47	5 (3-6)
**Sex**		
Female	673	**61 (58-64)**
Male	429	39 (36-42)
**Age groups in years**		
[1–10) years	288	**26 (24-29)**
[10–20) years	255	23 (21-26)
[20–30) years	189	17 (15-19)
[30–40) years	117	11 (9-13)
[40–60) years	162	15 (13-17)
[60–95] years	91	8 (7-10)

### 3.3 SARS-CoV-2 seroprevalence

**Fig 4:**
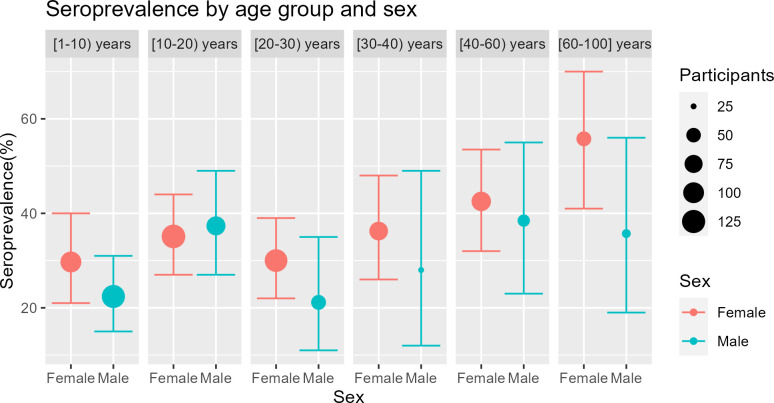
Seroprevalence by age and sex in Tombouctou.

A total of 309 participants tested seropositive out of 923 tested, yielding a crude seroprevalence of 33.5% (95% CI: 30.4% to 36.6%) (Fig 4).

It seems that the seroprevalence of COVID-19 increases with the age of the participants.

### 3.4 Projected number of cases and expected deaths based on seroprevalence data

Based on the age- and sex-specific seroprevalence estimates, we calculated the expected number of cases in the population of Tombouctou (70000 inhabitants). According to our estimates, the total number of infections that occurred from the start of the epidemic up until the survey date (January 2021) amounted to 22295 cases, including all ages and sexes (Table 2). During this same period, official COVID-19 situation reports in Mali recorded only 598 confirmed cases in the population of Tombouctou [39]. This discrepancy suggests an adjusted seroprevalence by age and sex of approximately 31.8% (95% CI: 31.5% to 32.2%), contrasting with an observed prevalence of only 0.8% (95% CI: 0.7% to 0.9%).

**Table 2:** Estimation of COVID-19 cases and deaths in the Tombouctou population (N = 1102).

		Prevalence (%)
		(95% CI)
**Serology**		
Positive	309	33.5% (30.4% to 36.6%)
Négative	614	
**Population (inhabitants in 2020)**	70 000	
**Infection**		
Cases, reported	598	0.80%
Estimated infections	22 295	31.8% (31.5% to 32.2%)
**Mortality**		
Deaths, reported	9	0.01
Deaths, estimated based on infections	156	0.2% (0.002 to 0.003)

Based on age- and sex-specific case-fatality rates reported at the start of the pandemic (February-March 2020 in China, before clinical management optimization), we estimated that approximately 156 of COVID-19 deaths may have occurred in the Tombouctou population (Table 2) from the beginning of the pandemic until the survey date (January 2021). In comparison, the officially reported number of deaths was only 9 [39].

### 3.5 Household living conditions

#### 3.5.1 Household profiles.

The HAC based on MCA enabled us to establish three household profiles (Table 3), differentiated by the following characteristics:

**Table 3:** Main Characteristics of the Obtained Classes (Household Profiles).

	TWH	LWH	MWH	p value	Subgroup analysis	

	(%)	(%)	(%)	Global	TWH vs LWH	TWH vs MWH
**Characteristics 1 (demographic indicator)**						
Household chief with low level of education (no school vs education)	100	88	64	<0.001*	0.347	0.001*
**Characteristic 2 (Traditional wealth)**						
Large household (≥10 people vs < 10 people)	47	9.5	18	0.004*	0.011*	0.002*
Goods: Livestock (yes vs no)	73	6.8	7	<0.001*	<0.001*	<0.001*
**Characteristic 3 (Modern goods and amenities)**						
Electricity (yes vs no)	0	12	96	<0.001*	0.347	<0.001*
Television (yes vs no)	0	16	86	<0.001*	0.206	<0.001*
Housing size (≥4 vs < 4 rooms)	73	70	96	<0.001*	1	0.003*
Access to water: running water at home (yes vs no)	0	36	89	<0.001*	0.004*	<0.001*

TWH: Traditionally Wealth Households, LWH: Low-Wealth Households, MWH: Modernly Wealth Households, *Significant after Bonferroni correction, Fisher’s exact test (p-value).

The first profile identified was that of “Traditionally Wealth Households” (TWH, n = 15). This profile is characterized by a very high level of traditional wealth [47% of households had more than 10 members, and the majority of these households owned livestock (73%)]. However, this profile shows a low level of modern goods and equipment: none of the households had access to electricity, a television, or running water.

The second profile identified was that of “Low-Wealth Households” (LWH, n = 74). This profile is characterized by low traditional wealth and few modern goods and equipment. It includes only 9.5% of households with more than 10 members, and 6.8% of households owning livestock. Furthermore, only 16% own a television, 12% have access to electricity, and 36% have access to water.

The third and final profile stands out due to significantly higher levels of modern goods and equipment (96% with electricity in the household, 86% with a television in the household, 89% with running water at home). As a result, this profile was labeled “Modernly Wealth Households” (MWH, n = 330). However, this profile has a lower proportion of traditional wealth, particularly livestock (7% vs. 73%, p < 0.001*) relative to the TWH profile. It is also associated with a smaller household size, with only 18% of large household.

### 3.6 Knowledge, Attitudes, Behaviors, and Practices (KABP)

The average knowledge score, measured using the 13 items (true/false/don’t know questions), showed no significant difference by gender, with an average of 9.0 (95% CI 6.0 to 10.0) for women and 9.0 (95% CI 7.0 to 10.0) for men (p > 0.9) (S2 Table). A significant proportion believed that COVID-19 was a divine punishment (39%), or that it was caused by witchcraft (21%) (S3 Table). Daily handwashing was reported as the most frequently adopted measure by participants: only 6.3% of participants indicated that they never washed their hands daily (S4 Table). A significant proportion of participants reported engaging in risky practices over the past 7 days: 37.8% never wear a mask outdoors, and 53.5% often visit crowded places during the day (S5 Table).

#### 3.6.1 KABP profiles.

The HAC based on MCA allowed us to establish the link between knowledge/attitudes and practices. Based on this link, we identified three at-risk groups (KABP profile) (Table 4), which are well distinguished by their level of knowledge (low, medium, high), which can, to some extent, explain their individual level of exposure to COVID-19.

**Table 4:** Main Characteristics of the Obtained Classes (KABP Profiles).

	PIP	MIM	INO	p value Global	Subgroup analysis	
	(%)	(%)	(%)		PIP vs MIM	PIP vs INO
**Characteristic 1 (Knowledge on COVID-19)**						
symptoms (yes vs no)	16	57	90	<0.001*	<0.001*	<0.001*
**Characteristic 2 (Risky Practices)**						
Wearing mask outside (never vs often)	49	2.9	0.5	<0.001*	<0.001*	<0.001*
**Characteristic 3 (Behavior changes)**						
Avoiding seeing friends (often vs never)	36	21	79	<0.001*	0.03	<0.001*
**Characteristic 4 (Attitudes/Opinions)**						
Divine punishment (strongly agree vs strongly disagree)	6.8	1.4	33	<0.001*	0.027	<0.001*
Introduced the white people (strongly agree vs strongly disagree)	4.9	1.7	29	<0.001*	0.031	0.011*

PIP: Poorly Informed and not Proactive in prevention, MIM: Moderately Informed and Moderately cautious, INO: Informed and cautious but with a Negative Opinion, *Significant after Bonferroni correction, Fisher’s exact test (p value).

The first identified profile, labeled “Poorly Informed and not Proactive in prevention” (PIP, n = 103), includes individuals with a low level of knowledge about COVID-19 (only 16% of them were aware of the symptoms of COVID-19), preventive practices inadequate (49% never wearing a mask outdoors) and behavioral changes moderate (36% having avoided seeing friends).

The second identified profile, labeled “Moderately Informed and Moderately cautious” (MIM, n = 348), exhibited an intermediate level of knowledge about COVID-19 (57% had knowledge of the symptoms of COVID-19), good preventive practices (only 2.9% never wearing a mask outdoors), and moderate behavior changes (21% having avoided seeing friends).

The third and last profile, titled “Informed and cautious but with a Negative Opinion” (INO, n = 186), was characterized by a high level of knowledge (90% had knowledge of the symptoms of COVID-19), good preventive practices (only 0.5% never wore a mask outdoors), and significant behavior changes (79% had avoided seeing friends). However, this profile had a negative perception of COVID-19 (33% believing that COVID-19 was a divine punishment and 29% thinking it had been imported to Mali by «white people»).

### 3.7 Factors associated with SARS-CoV-2 seropositivity

Participants living in households with more than one positive case were significantly associated with an increased likelihood of seropositivity [IRR = 5.47; 95% CI (4.51 to 6.64)], regardless of the household profile (Table 5). TWH and MWH were significantly associated with an increased risk of seropositivity compared to LWH, showing IRRs of 2.02 [95% CI (1.23 – 3.33)] and 1.33 [95% CI (1.02 – 1.72)], respectively (Table 5).

**Table 5:** Factors associated with SARS-CoV-2 seropositivity (Quasi-Poisson Model, n = 419).

Factors associated	IRR^1^	95% CI^1^	p-valeur
**Household profiles**			
MWH	1.33	1.02 – 1.72	**0.034**
TWH	2.02	1.23 – 3.33	**0.006**
LWH	—	—	
**Intra-household contamination**			
Yes	5.47	4.51 – 6.64	**<0.001**
No	—	—	

^1^ **IRR = incidence rate ratio, CI = confidence interval.**

*TWH: Traditionally Wealth Households, LWH: Low-Wealth Households, MWH: Modernly Wealth Households*.

Older individuals were significantly associated with an increased likelihood of seropositivity, with an adjusted odds ratio [AOR (95% CI)] of 1.02 (1.00 to 1.03) (Table 6). Women appeared to have an increased risk of seropositivity [not statistically significant, p-value = 0.2 with an adjusted odds ratio of 1.32 (0.86 to 2.03)]. Participants who were «Moderately Informed and Moderately cautious, MIM» and those who were «Informed and cautious but with a Negative Opinion, INO» regarding COVID-19 also seemed to have an increased risk of seropositivity (but not statistically significant) with adjusted odds ratios [AOR (95% CI)] of 1.19 (0.68 to 2.08) and 1.60 (0.88 to 2.91), compared to participants who were «Poorly Informed and not Proactive in prevention, PIP» (Table 6).

**Table 6:** Factors associated with SARS-CoV-2 seropositivity (GAMM Model, n = 554).

Factors associated	AOR^1^	95% CI^1^	p-value
**Sex**			
Female	1.32	0.86 – 2.03	0.2
Male	—	—	
**Age**	1.02	1.00 – 1.03	**0.005**
**KABP profiles**			
INO	1.19	0.68 – 2.08	0.5
MIM	1.60	0.88 – 2.91	0.12
PIP	—	—	

^1^ **AOR = adjusted odds ratio, CI = confidence interval.**

*PIP: Poorly Informed and not Proactive in prevention, MIM: Moderately Informed and Moderately cautious, INO: Informed and cautious but with a Negative Opinion.*

### 3.8 Geospatial analysis

#### 3.8.1 Mapping of seroprevalence.

**Fig 5:**
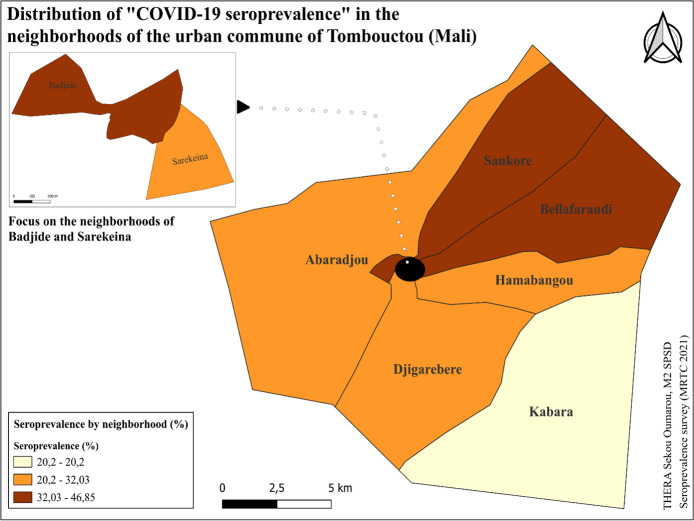
Seroprevalence of COVID-19 in the neighborhoods of Tombouctou.

The seroprevalence in the neighborhoods ranged from 20% to 47% (Fig 5). Therefore, we can identify three groups:

**Group 1 (High seroprevalence):** Neighborhoods of Sankore, Bellafarandi, and Badjide.

**Group 2 (Intermediate seroprevalence):** Neighborhoods of Abaradjou, Djigarebere, Hamabangou and Sarekeina.

**Group 3 (Low seroprevalence):** Neighborhoods of Kabara, which have lower rates compared to the neighborhoods in Group 1.

#### 3.8.2 Household profile mapping.

**Fig 6:**
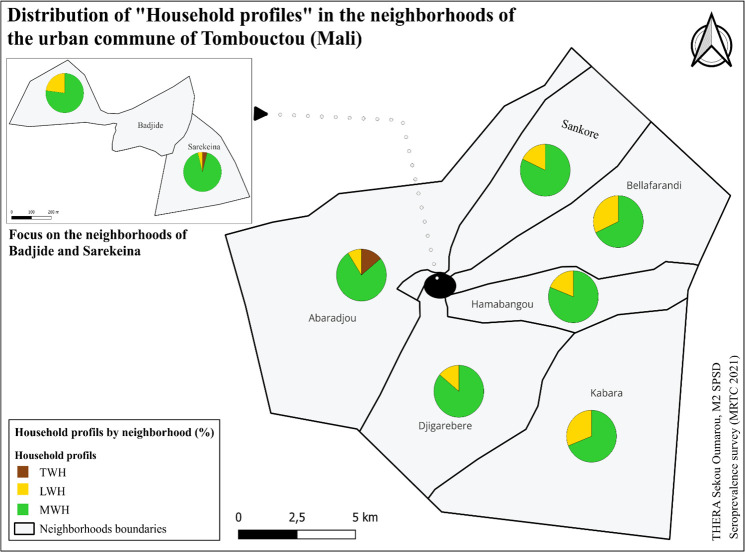
Household profiles in the neighborhoods of Tombouctou.

The TWH profile was found only in the neighborhoods of Abaradjou and Sarekeina (Fig 6). The LWH and MWH profiles were present in all neighborhoods with varying proportions.

#### 3.8.3 KABP profile mapping.

**Fig 7:**
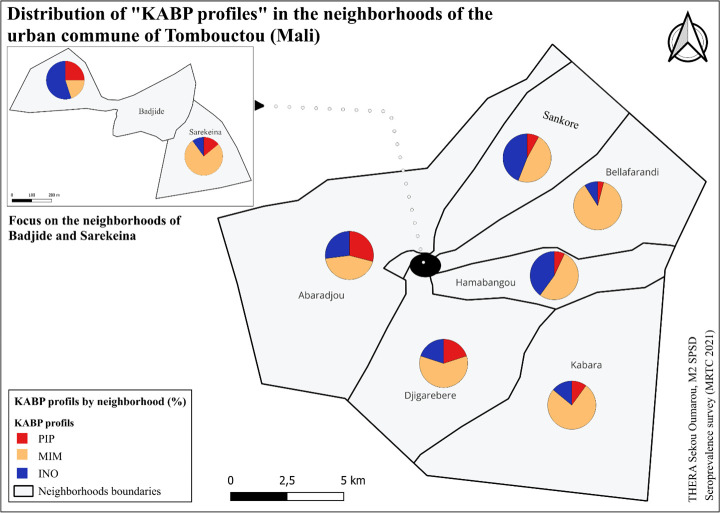
KABP profiles in the neighborhoods of Tombouctou.

The PIP profile was primarily found in the neighborhoods of Abaradjou, Badjide, and Djigarebere (Fig 7). The MIM profile was mostly present in the neighborhoods of Sarekeina, Hamabangou, Bellafarandi and Kabara. The INO profile was mainly found in the neighborhoods of Abaradjou, Hamabangou, Sankore, and Badjide.

#### 3.8.4 High-risk clusters.

We conducted a purely spatial analysis using SaTScan software with the Bernoulli model. The statistical unit was households. Cases and controls were defined as the number of positive and negative cases in each household, respectively.

**Fig 8:**
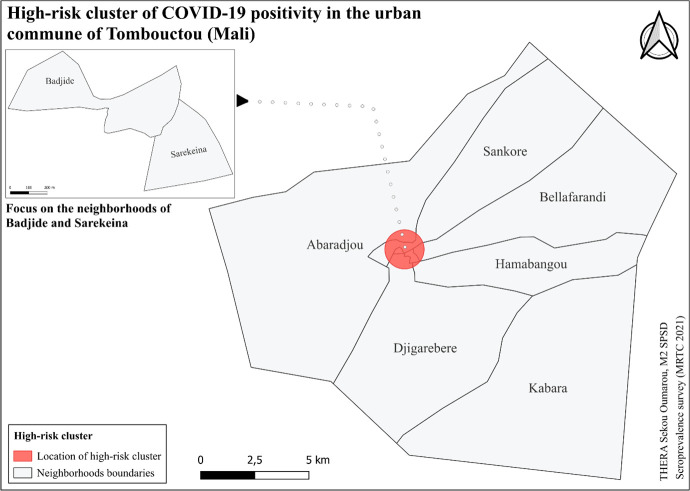
High-risk cluster identified in the neighborhoods of the urban commune of Tombouctou.

The analysis identified a high-risk cluster (coordinates: 16.775712 N, 3.008475 W) with a relative risk (RR) of 1.60 (p = 0.0031) and a population of 699 inhabitants, including 189 observed cases against 153 expected cases, within a radius of 0.91 km. This cluster is primarily concentrated in the neighborhoods of Sankore, Badjide, and Sarekeina (Fig 8).

## 4. Discussion

This study characterizes the spatial distribution and determinants of COVID-19 seroprevalence in an area with limited access to healthcare in Mali. It highlights active circulation of the virus, showing that participants living in traditionally or modernly wealth households, and older age are the most affected.

We estimated the R0 of the COVID-19 infection to be 2.08 [1.46-2.93]. This value is consistent with R0 estimates found in the literature [40,41] and those reported by the WHO [42], where values range from 2 to 3 and 1.4 to 2.5, respectively. Our estimate is lower than that reported in China during a meta-analysis, which was 3.3 [24]. We emphasize that our R0 may be underestimated as it was calculated based on the number of reported cases. It should be noted also that the severity of COVID-19 depends on age and comorbidities [43]. The population of Tombouctou is relatively young (41% are under 40 years old), and there are few elderly individuals with comorbidities. One factor that can affect the R0 is the level of access to healthcare, which is often difficult due to the security situation in the region. The low number of symptomatic or severe cases also limits the tendency to get tested. However, our estimate (R0 > 1) highlights the need to implement interventions to control the spread of the infection in this population [44].

The seroprevalence of SARS-CoV-2 in the urban commune of Tombouctou was 33.5% (95% CI: 30.4% to 36.6%), far exceeding the reported incidence of 0.8% during the same period (January 2021). At the city-wide level, this seroprevalence would correspond to 22295 cases, which is about one-third of the population of Tombouctou. Mortality projections suggest that deaths caused by COVID-19 are also underreported, with 9 declared deaths compared to approximately 156 expected. These results are in line to findings from South Sudan [45], Sierra Leone [46], and Afghanistan [47], where the number of implied infections were dozens of times higher than official counts. These findings underscore the limitations of routine surveillance in hard-to-reach areas and highlight the value of seroprevalence studies for revealing the true burden of disease. It appears that the seroprevalence increases with age among the study participants. Old females (≥ 60 years) reported a higher seroprevalence than old males (≥ 60 years). The higher level of SARS-CoV-2 seroprevalence in older females may be linked to their stronger connections with family members, friends, neighbors, and the broader community [48]. However, it is important to note that the number of adult men included in the study was small, which could result in a lower positivity rate.

The seroprevalence in the neighborhoods of the urban commune of Tombouctou varied from 20% to 47%. Thus, we identified three groups: low seroprevalence in the neighborhood of Kabara, intermediate seroprevalence in the neighborhoods of Abaradjou, Djigarebere, Hamabangou, and Sarekeina, and high seroprevalence in the neighborhoods of Sankore, Bellafarandi, and Badjide. This variation could be linked to the specific characteristics of each neighborhood. For example, the neighborhoods of Sankore, Bellafarandi, and Badjide, located in the city center, are the most densely populated and host important public places such as the main market, the Peace Monument, and Sankoré Square. In contrast, the neighborhood of Kabara is relatively distant from the city center, about 15 km away, and has a lower population density. These specificities could have influenced the dynamics of the COVID-19 pandemic in Tombouctou, underscoring the need to adapt public health interventions to local characteristics.

Living in a household where someone had been diagnosed with COVID-19, traditionally and modernly wealth households, and older age remain significantly associated with seropositivity, after adjusting for other available demographic and geographic covariates. These results are consistent with those of several studies.

A study conducted in China by Qin-Long Jing in 2020 showed that members of a household with a positive case of COVID-19 had an increased risk of contracting the virus due to proximity and prolonged contact with the infected person [49]. Households are deleterious environments for transmission, making it necessary to adapt specific prevention strategies for households, such as increased mask-wearing at home, availability and use of disinfectants within households, as well as health mediation actions to explain and adapt these prevention measures [50].

The epidemiological trends observed during the epidemic indicated that older people had higher infection rates and more severe health outcomes compared to younger people [51]. This can be explained by several factors, including the increased vulnerability of older adults due to a reduced effectiveness of immunity, as well as the presence of comorbidities [52]. These findings underscore the need to adapt epidemic control measures, prioritizing older individuals.

Participants living in traditionally wealth households and modernly wealth households had a higher risk of seropositivity compared to those living in low-wealth households. These results accord with those of a similar study conducted in Bamako, Mali, where the authors observed that high-income households had an increased likelihood of seropositivity (but not significant) compared to low-income households [10]. This concordance reflects the similarity in the socio-economic contexts between the two populations, suggesting that the determinants of seropositivity may be influenced by local factors such as wealth household, living conditions, access to healthcare, and preventive behaviors. These results differ from those reported in the southern region of France, where the highest incidence rates of SARS-CoV-2 were observed in areas with disadvantaged and very disadvantaged sociodemographic profiles [53]. In the United States, Zheng Li et al. also found that people living in areas with high socio-economic vulnerability had an increased probability of seropositivity [54]. Indeed, in the Malian context, people from wealth households may have greater mobility and more frequent social interactions, increasing their exposure to the virus [55,56]. Their households often host visitors or extended family members. Each of these factors may have contributed to the higher levels of seropositivity observed in our study.

We did not observe a significant link between behavioral profiles (KABP profiles) and seropositivity. This could be attributed to a statistical power issue, but also to the importance of the surrounding and middle level social context (familial, community…) which may influence household members’ health behaviors more than individual knowledge or societal level intervention such as governmental health policies. Even if participants are aware of the modes of transmission and prevention, it remains challenging to effectively protect themselves in the context of Tombouctou. A good understanding of the disease does not guarantee sufficient protection [57] and local community-based interventions have to be enhanced or implemented.

Our study has limitations. The sample size was underestimated due to a lack of available baseline data at the time of the survey, which may affect the generalizability of results. The household selection method was applied to reduce the geographical bias, but was not able to completely eliminate selection bias, potentially affecting the socio-economic and demographic diversity of the sample. As a cross-sectional survey, the study captured data at a single point in time, preventing conclusions about causality or changes over time. Moreover, the study relied on self-reported data, which are subject to recall bias. However, the cross-sectional survey was conducted in multiple stages to minimize potential biases.

## 5. Conclusion

Our analysis highlighted the active circulation of SARS-CoV-2 in Tombouctou, with higher seroprevalence observed among people from both traditionally and modernly wealth households, as well as older age groups. The findings underscore the need for tailored and targeted approach focusing on specific households, demographics and settings.

## Supporting information

S1 Data Time series of reported cases of COVID-19 in Tombouctou. (CSV)

S1 Checklist Questionnaire on inclusivity in global research. (DOCX)

S2 Data Individual-level data of study participants. (XLSX)

S1 Table Detailed serological results. (DOCX)

S2 Table Participants’ knowledge about COVID-19. (DOCX)

S3 Table Attitudes and opinions regarding COVID-19. (DOCX)

S4 Table Participants’ behaviors regarding COVID-19. (DOCX)

S5 Table Risky practices of participants related to COVID-19. (DOCX)

## References

[1] Morens DM, Daszak P, Markel H, Taubenberger JK. Pandemic COVID-19 joins history’s pandemic legion. mBio. 2020;11(3):e00812-20. doi: 10.1128/mBio.00812-20 32471830 PMC7267883

[2] Castro RR, Santos RSC, Sousa GJB, Pinheiro YT, Martins RRIM, Pereira MLD, et al. Spatial dynamics of the COVID-19 pandemic in Brazil. Epidemiol Infect. 2021;149:e60. doi: 10.1017/S0950268821000479 33629938 PMC7985898

[3] Tso FY, Lidenge SJ, Peña PB, Clegg AA, Ngowi JR, Mwaiselage J, et al. High prevalence of pre-existing serological cross-reactivity against severe acute respiratory syndrome coronavirus-2 (SARS-CoV-2) in sub-Saharan Africa. Int J Infect Dis. 2021;102:577–83. doi: 10.1016/j.ijid.2020.10.104 33176202 PMC7648883

[4] Dhama K, Khan S, Tiwari R, Sircar S, Bhat S, Malik YS, et al. Coronavirus disease 2019-COVID-19. Clin Microbiol Rev. 2020;33(4):e00028-20. doi: 10.1128/CMR.00028-20 32580969 PMC7405836

[5] Doumbia S, Sow Y, Diakite M, Lau C-Y. Coordinating the research response to COVID-19: Mali’s approach. Health Res Policy Syst. 2020;18(1):105. doi: 10.1186/s12961-020-00623-8 32943078 PMC7495403

[6] Sagara I, Woodford J, Kone M, Assadou MH, Katile A, Attaher O, et al. Rapidly increasing SARS-CoV-2 seroprevalence and limited clinical disease in three Malian communities: a prospective cohort study. medRxiv. 2021:2021.04.26.21256016. doi: 10.1101/2021.04.26.21256016 34185847 PMC8394825

[7] Mali: WHO coronavirus disease (COVID-19) dashboard with vaccination data. [cited 2023 Nov 28]. Available from: https://covid19.who.int

[8] Etang-Ndip A, Hoogeveen J, Lendorfer J. Impact socio-économique de la crise au nord du Mali sur les personnes déplacées. Rapp Rech Banq Mond; 2015 [cited 2024 Jan 17]. Available from: https://documents.worldbank.org/curated/en/598931467999718012/pdf/Impact0socio0000personnes0d0plac0es.pdf

[9] Direction régional de la santé. Rapport de situation de covid-19 dans la région de Tombouctou (Mali). Mali; 2020.

[10] Cissoko M, Landier J, Kouriba B, Sangare AK, Katilé A, Djimde AA, et al. SARS-CoV-2 seroprevalence and living conditions in Bamako (Mali): a cross-sectional multistage household survey after the first epidemic wave, 2020. BMJ Open. 2023;13(4):e067124. doi: 10.1136/bmjopen-2022-067124 37080622 PMC10123860

[11] Temessadouno FW, Ndong JG, Gignoux E, Coppieters Y, Ba A, Sidibe YD, et al. Seroprevalence of anti-SARS-CoV-2 antibodies among blood donors from December 2020 to June 2021 in Koutiala district, Mali. PLOS Glob Public Health. 2023;3(1):e0001316. doi: 10.1371/journal.pgph.0001316 36962828 PMC10022217

[12] Daw MA. The impact of armed conflict on the epidemiological situation of COVID-19 in Libya, Syria and Yemen. Front Public Health. 2021;9:667364. doi: 10.3389/fpubh.2021.667364 34178925 PMC8226094

[13] Daw MA, El-Bouzedi AH, Ahmed MO. The epidemiological and spatiotemporal characteristics of the 2019 novel coronavirus disease (COVID-19) in Libya. Front Public Health. 2021;9:628211. doi: 10.3389/fpubh.2021.628211 34195168 PMC8236517

[14] Daw MA, El-Bouzedi AH, Ahmed MO, Alejenef AA. The epidemiological characteristics of COVID-19 in Libya during the ongoing-armed conflict. Pan Afr Med J. 2020;37:219. doi: 10.11604/pamj.2020.37.219.24993 33520058 PMC7821789

[15] Ekzayez A, Alhaj Ahmad Y, Alhaleb H, Checchi F. The impact of armed conflict on utilisation of health services in north-west Syria: an observational study. Confl Health. 2021;15(1):91. doi: 10.1186/s13031-021-00429-7 34906188 PMC8670035

[16] Lucero-Prisno DE 3rd, Essar MY, Ahmadi A, Lin X, Adebisi YA. Conflict and COVID-19: a double burden for Afghanistan’s healthcare system. Confl Health. 2020;14:65. doi: 10.1186/s13031-020-00312-x 32973920 PMC7506810

[17] Fatima M, O’Keefe KJ, Wei W, Arshad S, Gruebner O. Geospatial analysis of COVID-19: a scoping review. Int J Environ Res Public Health. 2021;18(5):2336. doi: 10.3390/ijerph18052336 33673545 PMC7956835

[18] Mollalo A, Mohammadi A, Mavaddati S, Kiani B. Spatial analysis of COVID-19 vaccination: a scoping review. Int J Environ Res Public Health. 2021;18(22):12024. doi: 10.3390/ijerph182212024 34831801 PMC8624385

[19] Franch-Pardo I, Napoletano BM, Rosete-Verges F, Billa L. Spatial analysis and GIS in the study of COVID-19. A review. Sci Total Environ. 2020;739:140033. doi: 10.1016/j.scitotenv.2020.140033 32534320 PMC7832930

[20] Ramírez-Aldana R, Gomez-Verjan JC, Bello-Chavolla OY. Spatial analysis of COVID-19 spread in Iran: Insights into geographical and structural transmission determinants at a province level. PLoS Negl Trop Dis. 2020;14(11):e0008875. doi: 10.1371/journal.pntd.0008875 33206644 PMC7710062

[21] Islam N, Lacey B, Shabnam S, Erzurumluoglu AM, Dambha-Miller H, Chowell G, et al. Social inequality and the syndemic of chronic disease and COVID-19: county-level analysis in the USA. J Epidemiol Community Health. 2021;75:496–500. doi: 10.1136/jech-2020-215626 33402397

[22] Fielding-Miller RK, Sundaram ME, Brouwer K. Social determinants of COVID-19 mortality at the county level. Epidemiology. 2020. doi: 10.1101/2020.05.03.20089698 PMC7556498 33052932

[23] World Health Organization. Population-based age-stratified seroepidemiological investigation protocol for COVID-19 virus infection, 17 March 2020. World Health Organization; 2020. Report No.: WHO/2019-nCoV/Seroepidemiology/2020.1. Available from: https://apps.who.int/iris/handle/10665/331656

[24] Alimohamadi Y, Taghdir M, Sepandi M. Estimate of the basic reproduction number for COVID-19: a systematic review and meta-analysis. J Prev Med Public Health. 2020;53(3):151–7. doi: 10.3961/jpmph.20.076 32498136 PMC7280807

[25] Nikbakht R, Baneshi MR, Bahrampour A, Hosseinnataj A. Comparison of methods to estimate basic reproduction number (R0) of influenza, using Canada 2009 and 2017-18 A (H1N1) data. J Res Med Sci. 2019;24:67. doi: 10.4103/jrms.JRMS_888_18 31523253 PMC6670001

[26] Prada JP, Maag LE, Siegmund L, Bencurova E, Liang C, Koutsilieri E, et al. Estimation of R0 for the spread of SARS-CoV-2 in Germany from excess mortality. Sci Rep. 2022;12(1):17221. doi: 10.1038/s41598-022-22101-7 36241688 PMC9562071

[27] Elsaid M, Nasef MA, Huy NT. R0 of COVID-19 and its impact on vaccination coverage: compared with previous outbreaks. Hum Vaccin Immunother. 2021;17(11):3850–4. doi: 10.1080/21645515.2020.1865046 34612165 PMC8827628

[28] Wu JT, Leung K, Leung GM. Nowcasting and forecasting the potential domestic and international spread of the 2019-nCoV outbreak originating in Wuhan, China: a modelling study. Lancet. 2020;395(10225):689–97. doi: 10.1016/S0140-6736(20)30260-9 32014114 PMC7159271

[29] Institut National de la Statistique (INSTAT)-Mali. 2020. Available from: https://mali.opendataforafrica.org/

[30] Bennett S, Radalowicz A, Vella V, Tomkins A. A computer simulation of household sampling schemes for health surveys in developing countries. Int J Epidemiol. 1994;23(6):1282–91. doi: 10.1093/ije/23.6.1282 7721532

[31] Biomérieux SA. BioMérieux | VIDAS® SARS-COV-2. [cited 2024 Jun 10]. Available from: https://go.biomerieux.com/SEROLOGIE-VIDAS-SARS-COV-2

[32] Garenne M. Traditional wealth, modern goods, and demographic behavior in rural Senegal. World Dev. 2015;72:267–76. doi: 10.1016/j.worlddev.2015.03.013

[33] The Novel Coronavirus Pneumonia Emergency Response Epidemiology Team. The epidemiological characteristics of an outbreak of 2019 novel coronavirus diseases (COVID-19) - China, 2020. China CDC Wkly. 2020;2(8):113–22. doi: 10.46234/ccdcw2020.032 34594836 PMC8392929

[34] Lê S, Josse J, Husson F. FactoMineR: an R package for multivariate analysis. J Stat Soft. 2008;25(1):1–18. doi: 10.18637/jss.v025.i01

[35] Gelb PA et J. Introduction | Méthodes quantitatives en sciences sociales: un grand bol d’R. Available from: https://laeq.github.io/LivreMethoQuantBolR/sect111.html

[36] Wood SN. Modèles additifs généralisés: une introduction avec R.

[37] Bherwani H, Anjum S, Kumar S, Gautam S, Gupta A, Kumbhare H, et al. Understanding COVID-19 transmission through Bayesian probabilistic modeling and GIS-based Voronoi approach: a policy perspective. Environ Dev Sustain. 2021;23(4):5846–64. doi: 10.1007/s10668-020-00849-0 32837277 PMC7340861

[38] Kulldorff M. A spatial scan statistic. Commun Stat Theory Methods. 1997;26(6):1481–96. doi: 10.1080/03610929708831995

[39] Government of the Republic of Mali. Rapport de situation COVID-19 au Mali, 20 mars 2022/ N°191 – Mali; 2022. Available from: https://reliefweb.int/report/mali/rapport-de-situation-covid-19-au-mali-20-mars-2022-n-191

[40] Liu Y, Gayle AA, Wilder-Smith A, Rocklöv J. The reproductive number of COVID-19 is higher compared to SARS coronavirus. J Travel Med. 2020;27(2):taaa021. doi: 10.1093/jtm/taaa021 32052846 PMC7074654

[41] Musa SS, Zhao S, Wang MH, Habib AG, Mustapha UT, He D. Estimation of exponential growth rate and basic reproduction number of the coronavirus disease 2019 (COVID-19) in Africa. Infect Dis Poverty. 2020;9(1):96. doi: 10.1186/s40249-020-00718-y 32678037 PMC7365306

[42] Statement on the meeting of the International Health Regulations (2005) Emergency Committee regarding the outbreak of novel coronavirus 2019 (n-CoV) on 23 January 2020. [cited 2024 Jun 13]. Available from: https://www.who.int/news/item/23-01-2020-statement-on-the-meeting-of-the-international-health-regulations-(2005)-emergency-committee-regarding-the-outbreak-of-novel-coronavirus-(2019-ncov)

[43] Mohan AA, Olson LB, Naqvi IA, Morrison SA, Kraft BD, Chen L, et al. Age and comorbidities predict COVID-19 outcome, regardless of innate immune response severity: a single institutional cohort study. Crit Care Explor. 2022;4(12):e0799. doi: 10.1097/CCE.0000000000000799 36506827 PMC9726311

[44] Kwok KO, Tang A, Wei VWI, Park WH, Yeoh EK, Riley S. Epidemic models of contact tracing: systematic review of transmission studies of severe acute respiratory syndrome and middle east respiratory syndrome. Comput Struct Biotechnol J. 2019;17:186–94. doi: 10.1016/j.csbj.2019.01.003 30809323 PMC6376160

[45] Wiens KE, Mawien PN, Rumunu J, Slater D, Jones FK, Moheed S, et al. Seroprevalence of severe acute respiratory syndrome coronavirus 2 IgG in Juba, South Sudan, 20201. Emerg Infect Dis. 2021;27(6):1598–606. doi: 10.3201/eid2706.210568 34013872 PMC8153877

[46] Barrie MB, Lakoh S, Kelly JD, Kanu JS, Squire JS, Koroma Z, et al. SARS-CoV-2 antibody prevalence in Sierra Leone, March 2021: a cross-sectional, nationally representative, age-stratified serosurvey. BMJ Glob Health. 2021;6(11):e007271. doi: 10.1136/bmjgh-2021-007271 34764148 PMC8587532

[47] Saeedzai SA, Sahak MN, Arifi F, Abdelkreem Aly E, Gurp M van, White LJ, et al. COVID-19 morbidity in Afghanistan: a nationwide, population-based seroepidemiological study. BMJ Open. 2022;12(7):e060739. doi: 10.1136/bmjopen-2021-060739 35896297 PMC9334691

[48] Cornwell B. Independence through social networks: bridging potential among older women and men. J Gerontol B Psychol Sci Soc Sci. 2011;66(6):782–94. doi: 10.1093/geronb/gbr111 21983039 PMC3198249

[49] Jing Q-L, Liu M-J, Zhang Z-B, Fang L-Q, Yuan J, Zhang A-R, et al. Household secondary attack rate of COVID-19 and associated determinants in Guangzhou, China: a retrospective cohort study. Lancet Infect Dis. 2020;20(10):1141–50. doi: 10.1016/S1473-3099(20)30471-0 32562601 PMC7529929

[50] Fruleux A, Gaudart J, Franke F, Nauleau S, Dutrey Kaiser A, Legendre E, et al. Reviving health mediation during the COVID-19 crisis and beyond: an implementation study in deprived neighbourhoods of Marseille, France. Front Public Health. 2024;12. doi: 10.3389/fpubh.2024.1313575 PMC11251881 39022414

[51] Clark A, Jit M, Warren-Gash C, Guthrie B, Wang HHX, Mercer SW, et al. Global, regional, and national estimates of the population at increased risk of severe COVID-19 due to underlying health conditions in 2020: a modelling study. Lancet Glob Health. 2020;8(8):e1003–17. doi: 10.1016/S2214-109X(20)30264-3 32553130 PMC7295519

[52] Rahman MM, Hamiduzzaman M, Akter MS, Farhana Z, Hossain MK, Hasan MN, et al. Frailty indexed classification of Bangladeshi older adults’ physio-psychosocial health and associated risk factors- a cross-sectional survey study. BMC Geriatr. 2021;21(1):3. doi: 10.1186/s12877-020-01970-5 33402094 PMC7786917

[53] Landier J, Bassez L, Bendiane M-K, Chaud P, Franke F, Nauleau S, et al. Social deprivation and SARS-CoV-2 testing: a population-based analysis in a highly contrasted southern France region. Front Public Health. 2023;11:1162711. doi: 10.3389/fpubh.2023.1162711 37250096 PMC10213643

[54] Li Z, Lewis B, Berney K, Hallisey E, Williams AM, Whiteman A, et al. Social vulnerability and rurality associated with higher severe acute respiratory syndrome coronavirus 2 (SARS-CoV-2) infection-induced seroprevalence: a nationwide blood donor study-United States, July 2020-June 2021. Clin Infect Dis. 2022;75(1):e133–43. doi: 10.1093/cid/ciac105 35137014 PMC8903418

[55] Nouvellet P, Bhatia S, Cori A, Ainslie KEC, Baguelin M, Bhatt S, et al. Reduction in mobility and COVID-19 transmission. Nat Commun. 2021;12(1):1090. doi: 10.1038/s41467-021-21358-2 33597546 PMC7889876

[56] Mossong J, Hens N, Jit M, Beutels P, Auranen K, Mikolajczyk R, et al. Social contacts and mixing patterns relevant to the spread of infectious diseases. PLoS Med. 2008;5(3):e74. doi: 10.1371/journal.pmed.0050074 18366252 PMC2270306

[57] Nwagbara UI, Osual EC, Chireshe R, Bolarinwa OA, Saeed BQ, Khuzwayo N, et al. Knowledge, attitude, perception, and preventative practices towards COVID-19 in sub-Saharan Africa: a scoping review. PLoS One. 2021;16(4):e0249853. doi: 10.1371/journal.pone.0249853 33872330 PMC8055009

