# Supplementary material for: Distribution and determinants of COVID-19 seroprevalence in a hard-to-access health district in Mali
Source: PLOS Glob Public Health. 2025 Jul 21;5(7):e0004842. doi: 10.1371/journal.pgph.0004842 (PMC12279100; doi:10.1371/journal.pgph.0004842)
Supplement: S1 Table — (DOCX) [file pgph.0004842.s004.docx]

**S1 Table. Detailed serological results**

| **Serological results** | **n** | **%** |
| --- | --- | --- |
| **COVID-19 serologye** | | |
| Negative | 614 | 55,7 |
| Positive | 309 | **28** |
| Not interpretable | 179 | 16,2 |
| **Total** | **1102** | **100** |
| **Interpretable serological test details** | | |
| Negative | 614 | 66,5 |
| IgG positive, IgM negative | 262 | 28,4 |
| IgG negative, IgM positive | 19 | 2,1 |
| IgG positive + IgM positive | 28 | 3 |
| **Total** | **923** | **100** |
