# Supplementary material for: Distribution and determinants of COVID-19 seroprevalence in a hard-to-access health district in Mali
Source: PLOS Glob Public Health. 2025 Jul 21;5(7):e0004842. doi: 10.1371/journal.pgph.0004842 (PMC12279100; doi:10.1371/journal.pgph.0004842)
Supplement: S2 Table — (DOCX) [file pgph.0004842.s005.docx]

**S2 Table. Participants' Knowledge about COVID-19**

| **Knowledge on COVID-19, (Tombouctou, N=637, Janvier 2021)** | |
| --- | --- |
| **Questions (true/false/don’t know)** | **Success (%)** |
| 1. The main clinical symptoms of the disease are dry cough, fever, tiredness and muscle pain. (true) | 77,7 |
| 2. Unlike the common cold, blocked nose, runny nose and sneezing are not symptoms that are systematically associated with COVID-19. (true) | 60,1 |
| 3. At present, there is no treatment for COVID-19 but early treatment of symptoms can help patients recover. (true) | 70,6 |
| 4. Not all infected people will develop severe forms of the disease. (true) | 65 |
| 5. Older people, those with chronic illnesses and the obese are at greater risk of developing a severe form. (true) | 72,1 |
| 6. Eating or touching wild animals can lead to infection. (false) | 14,9 |
| 7. People with the virus, if they do not have a fever, are not contagious and therefore cannot transmit COVID-19 to others. (false) | 16,2 |
| 8. COVID-19 is primarily transmitted by respiratory droplets from infected persons. (true) | 76,8 |
| 9. Residents of epidemic areas can wear masks to prevent infection by the COVID-19 virus. (true) | 69,1 |
| 10. COVID-19 prevention measures do not apply to children and young adults. (false) | 33,6 |
| 11. To prevent COVID-19 infection, people should avoid going to populated places (mosques, markets, railway stations). (true) | 78,5 |
| 12. Isolating infected people helps to reduce transmission of the virus. (true) | 68,3 |
| 13. Any person in contact with an infected person should be isolated in a suitable place for an observation period of 14 days. (true) | 85,1 |

|  | **Female, N = 455*^1^*** | **Male, N = 182*^1^*** | **p-value*^2^*** |
| --- | --- | --- | --- |
| **Mean score (SD)** | 9,0 (6,0 – 10,0) | 9,0 (7,0 – 10,0) | >0,9 |
| ***^1^* Mediane (IE)** | | | |
| ***^2^* Wilcoxon-Mann-Whitney test** | | | |
