# Supplementary material for: Distribution and determinants of COVID-19 seroprevalence in a hard-to-access health district in Mali
Source: PLOS Glob Public Health. 2025 Jul 21;5(7):e0004842. doi: 10.1371/journal.pgph.0004842 (PMC12279100; doi:10.1371/journal.pgph.0004842)
Supplement: S3 Table — (DOCX) [file pgph.0004842.s006.docx]

**S3 Table. Attitudes and Opinions Regarding COVID-19**

| **Attitudes, Opinions Regarding COVID-19** | **N = 637*^1^*** |
| --- | --- |
| **Is a God punishment** | |
| Strongly agreed | 73 (11%) |
| Agreed | **246 (39%)** |
| Don’t know | 70 (11%) |
| Neither agree nor disagree | 22 (3,5%) |
| Disagree | **180 (28%)** |
| Strongly Disagree | 46 (7,2%) |
| **Has been introduced in Mali by the white people** | |
| Strongly agreed | 65 (10%) |
| Agreed | **154 (24%)** |
| Don’t know | 112 (18%) |
| Neither agree nor disagree | 37 (5,8%) |
| Disagree | **240 (38%)** |
| Strongly Disagree | 29 (4,6%) |
| **Is due to a spell** | |
| Strongly agreed | 74 (12%) |
| Agreed | **132 (21%)** |
| Don’t know | 135 (21%) |
| Neither agree nor disagree | 41 (6,4%) |
| Disagree | **234 (37%)** |
| Strongly Disagree | 21 (3,3%) |
| **Help politicians’ strategy to take money from developed countries** | |
| Strongly agreed | 75 (12%) |
| Agreed | **138 (22%)** |
| Don’t know | 140 (22%) |
| Neither agree nor disagree | 23 (3,6%) |
| Disagree | **236 (37%)** |
| Strongly Disagree | 25 (3,9%) |
| ***^1^* n (%)** | |
