# Supplementary material for: Distribution and determinants of COVID-19 seroprevalence in a hard-to-access health district in Mali
Source: PLOS Glob Public Health. 2025 Jul 21;5(7):e0004842. doi: 10.1371/journal.pgph.0004842 (PMC12279100; doi:10.1371/journal.pgph.0004842)
Supplement: S4 Table — (DOCX) [file pgph.0004842.s007.docx]

**S4 Table. Participants' Behaviors Regarding COVID-19**

| **Participants' Behaviors Regarding COVID-19** | **N = 637**  **n (%)** |
| --- | --- |
| **Washing hands** | |
| Never | 40 (6,3%) |
| Often | **299 (46,9%)** |
| Systematically | **298 (46,8%)** |
| **Blowing into the elbow** | |
| Never | 83 (13%) |
| Often | **407 (63,9%)** |
| Systematically | **147 (23,1%)** |
| **Stop touching other people** | |
| Never | 177 (27,8%) |
| Often | **382 (60%)** |
| Systematically | **78 (12%)** |
| **Travelling less frequently** | |
| Never | 90 (14,2%) |
| Often | **474 (74,4%)** |
| Systematically | **73 (11,4%)** |
| **Avoiding populated places** | |
| Never | 81 (12,7%) |
| Often | **471 (74%)** |
| Systematically | **85 (13,3%)** |
| **Avoiding seeing friends** | |
| Never | 85 (13,7%) |
| Often | **479 (75,2%)** |
| Systematically | **71 (11,1%)** |
