# Supplementary material for: Distribution and determinants of COVID-19 seroprevalence in a hard-to-access health district in Mali
Source: PLOS Glob Public Health. 2025 Jul 21;5(7):e0004842. doi: 10.1371/journal.pgph.0004842 (PMC12279100; doi:10.1371/journal.pgph.0004842)
Supplement: S5 Table — (DOCX) [file pgph.0004842.s008.docx]

**S5 Table. Risky Practices of Participants Related to COVID-19**

| **Risky Practices of Participants Related to COVID-19** | **N = 637*^1^*** |
| --- | --- |
| **Wearing mask outside** | |
| Never | **241 (37,8%)** |
| Often | **360 (56%)** |
| Systematically | 36 (5,6%) |
| **Visiting populated public places on day** | |
| Never | **267 (41,9%)** |
| Often | **341 (53,5%)** |
| Every day | 29 (4,6%) |
| **Visiting populated public places on night** | |
| Never | **457 (72%)** |
| Often | **167 (26%)** |
| Every night | 13 (2%) |
| **Staying more than 2 hours in a small closed space** | |
| Never | **352 (52,2%)** |
| Often | **257 (40,3%)** |
| Systematically | 28 (4,4%) |
| ***^1^* n (%)** | |
